# Relationship Between Leptin and Heart Failure: A Meta-Analysis

**DOI:** 10.5334/gh.1434

**Published:** 2025-05-23

**Authors:** Xin Sun, Caihong Xin, Jiayi Yao, Hongli Wang

**Affiliations:** 1 Department of Endocrinology and Metabolism, The First Affiliated Hospital of Soochow University, Suzhou, CN; 2 Department of Endocrinology and Metabolism, The Fourth People’s Hospital of Soochow University, Shenyang, CN; 3 Department of Cardiology, The Second Affiliated Hospital of Dalian Medical University, Dalian, CN

**Keywords:** leptin, heart failure, HF

## Abstract

**Background:**

Heart failure (HF) is a diverse and potentially fatal condition affecting more than 60 million people worldwide. Previous studies have identified a close relationship between leptin levels and HF, and that leptin levels in patients with HF are higher than those in healthy individuals. However, some studies have reported inconsistent results. Therefore, the association between leptin levels and HF remains controversial.

**Methods:**

A literature search was conducted on the Web of Science, Wiley Online Library, Embase, and PubMed databases. The title or abstract search term ‘leptin’ was used in combination with ‘heart failure’ and ‘HF’. Meta-analysis results were reported as standardized mean differences (SMD) with corresponding 95% confidence intervals (CI).

**Results:**

Eighteen studies comprising 1149 patients with HF and 622 healthy controls were included in the meta-analysis. Leptin levels in patients with HF were significantly higher than those in healthy individuals (SMD, 0.54; 95% CI [0.15, 0.93]).

**Conclusions:**

To our knowledge, this systematic review is the first to evaluate the relationship between leptin and HF. Determining the role of leptin in HF will significantly contribute to its diagnosis and treatment.

## Introduction

Heart failure (HF) is a diverse and potentially fatal condition affecting more than 60 million people worldwide. It is associated with significant illness and death, reduced quality of life, and substantial strain on healthcare resources, including usage and costs (1). Despite pharmacological treatment, the annual HF mortality rate exceeds 20% (2). Hence, early diagnosis and prognostic evaluation of patients with HF, along with appropriate interventions, are of utmost significance. Obesity is widely acknowledged as an independent risk factor for HF (3). Nevertheless, epidemiological investigations have revealed that obese HF patients may have a better prognosis than those with normal weight (4,5), a phenomenon referred to as the ‘obesity paradox’ (6). The obesity paradox exists in multiple cardiovascular diseases, including myocardial infarction, hypertension, patients undergoing coronary artery bypass grafting, peripheral vascular diseases, atrial fibrillation, and aortic stenosis (7,8,9). Regardless of geographical setting, sex, age range, and presence of comorbidities, the medical community has reached a consensus that patients with HF and a higher BMI have relatively better prognoses (10). However, the mechanism underlying this paradoxical phenomenon remains unclear. It is thought that adipose tissue may play a crucial role. Adipose tissue has previously been regarded as an energy-storage organ; however, it is now considered an endocrine organ that secretes large quantities of adipokines, exerting effects on various organs and tissues.

Leptin, the first fat factor discovered, exists in particularly high levels in the blood of patients with obesity and myocardial infarction. Leptin primarily reduces cardiac output by exerting a negative inotropic effect on cardiomyocytes. It also regulates cardiac metabolism, cardiomyocyte morphology, and extracellular matrix production (11). Research has shown that the expression of transcriptional activator-3 (signal transducer and activator of transcription-3, STAT-3) and AMPK can be upregulated by leptin and its receptor in cardiomyocytes, thereby exerting cardioprotective effects on patients with HF by improving myocardial hypertrophy, reducing cell apoptosis, and controlling inflammatory responses (12).

Some studies reported that leptin levels in patients with HF were significantly lower than those in healthy individuals (13,14). However, the results of similar studies were inconsistent with those of earlier studies (15,16). Therefore, the relationship between leptin levels and HF remains controversial, and whether changes in leptin levels are related to HF remains unclear. Consequently, this meta-analysis aimed to systematically and comprehensively evaluate the relationship between leptin levels and HF.

## Methods

### Literature search

A literature search of the Web of Science, Wiley Online Library, PubMed, and Embase databases was performed. The search scope included studies investigating the relationship between leptin and patients with HF, up to October 2024. The title or abstract search term ‘leptin’ was used in combination with ‘heart failure’. Eligible studies were restricted to those published in English. The reference lists of retrieved studies were manually searched to identify additional potentially eligible studies. The study was registered in the PROSPERO Database (CRD42024594741). All requisite items reported for systematic reviews and meta-analyses are listed in the Supplementary Data (File S1). Full search strategy in databases is also provided in Supplementary Data (File S2).

### Inclusion criteria

The meta-analysis was performed on studies fulfilling the following criteria: sufficient data regarding leptin levels in patients with HF and healthy controls; a case-control design; and publication in English.

### Exclusion criteria

Duplicate studies, those with insufficient data, animal studies, meta-analyses, reviews, meeting summaries, case reports, and editorials were excluded.

### Data extraction and risk of bias

Two investigators (SX and YJY) independently reviewed the titles and abstracts of the retrieved studies according to predetermined inclusion and exclusion criteria. Disagreements regarding study selection during the review process were resolved through consensus discussion based on an established standard. If consensus could not be reached, a third investigator was invited to participate in the final decision as to whether the study fulfilled the inclusion criteria. Data extracted from the selected studies included the following: first author, publication year, research methods, sample size, age, sex, and population.

The Cochrane Collaboration recommends the Newcastle-Ottawa Scale (NOS) as a tool for assessing bias in observational studies (17). The NOS was used as an evaluation standard, and two researchers were based on the research content of the case-control studies and cohort studies, the methodological quality of independent evaluation, including selection of the research objective, comparability between groups, and outcome measures. An NOS score between 0 and 9 was calculated, with ≥6 points considered to be a high-quality study; the higher the score, the higher the quality of the study (18).

### Statistical analysis

The results are expressed as the standardized mean difference (SMD) with a corresponding 95% confidence interval (CI). Heterogeneity was assessed according to *I*^2^ and *P* values, as follows: *I*^2^ < 50% and *P* > 0.1 represented low heterogeneity and a fixed-effects model was used for analysis; *I*^2^ ≥ 50% and *P* ≤ 0.1 represented high heterogeneity and a random-effects model was used for analysis. Sensitivity analyses were performed for the sources of heterogeneity of the outcome indicators. Egger’s and Begger’s tests were used to assess publication bias. A *P* < 0.05 was considered statistically significant. Statistical analysis was performed using Stata 12.0 (StataCorp LLC, College Station, TX, USA).

## Results

### Literature search and study selection

**Figure 1:**
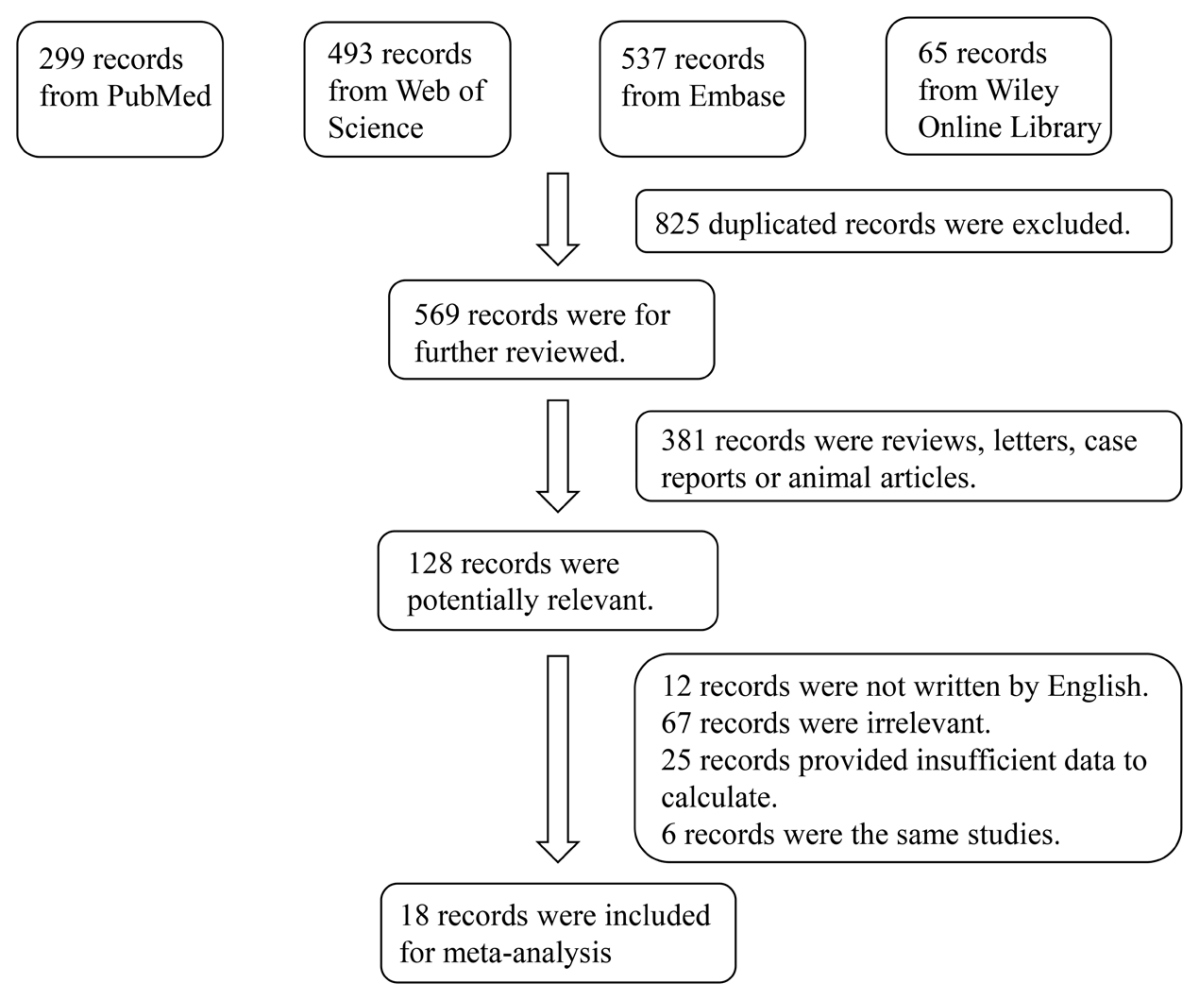
Flowchart of the detailed procedure for the inclusion or exclusion of selected studies.

In total, 1,394 relevant studies were retrieved from the Web of Science, Wiley Online Library, PubMed, and Embase databases. Finally, 18 studies comprising 1149 patients with HF and 622 healthy controls were included in the meta-analysis (13,14,15,16,19,20,21,22,23,24,25,26,27,28,29,30,31,32). A flow diagram illustrating the study selection process is shown in Figure 1. The characteristics of the included studies are summarized in Table 1. All 18 studies included in this meta-analysis fulfilled the criteria for the not otherwise specified (NOS) categories of selection, comparability, and exposure.

**Table 1:** Study characteristics of the published studies included in the meta-analysis.


AUTHOR	PUBLICATION YEAR	REGION	NUMBER		LEPTIN		DETAILS	
		
			CASE	CONTROL	CASE	CONTROL	CASE	CONTROL

Toth MJ	1997	USA	18	46	5.1 ± 4.2	6.8 ± 4.4	Eighteen patients (aged 71 ± 6 years) with HF were recruited. Ten patients with documented coronary artery disease and eight patients with dilated cardiomyopathy unrelated to coronary artery disease.	Forty-six healthy elderly subjects (aged 66 ± 6 years) served as a control group.

Leyva F	1998	Germany	25	18	8.12 ± 1.3	4.48 ± 0.7	The study group consisted of 25 patients with congestive HF. Chronic HF was secondary to coronary heart disease in 13 and to idiopathic dilated cardiomyopathy in 12.	Healthy controls were matched to congestive HF patients for age, sex, and total body fat.

Murdoch DR	1999	UK	51	26	10.82 ± 5.86	18.3 ± 3.5	The study group consisted of 51 patients, aged 52 to 83 years, with stable chronic HF of more than 3 months duration. The primary cause of HF was coronary artery disease in 48 patients and regurgitant valve disease in 3 patients.	The control group consisted of 26 matched healthy volunteers, aged 30 to 76 years.

El-Bindary EM	2001	Egypt	80	20	16.94 ± 6.33	12.25 ± 3.9	The study group consisted of 47 patients (aged 47 ± 4 years) with chronic HF.	The control group consisted of 20 aged- and sex-matched healthy volunteers.

Doehner W	2001	Germany	47	21	7.6 ± 0.7	4.8 ± 0.7	The study group consisted of 47 patients (aged 61 ± 2 years) with chronic HF due to ischemic heart disease (n = 28) or idiopathic dilated cardiomyopathy (n = 19).	The control group consisted of 21 healthy subjects (aged 59 ± 3 years) of similar age.

Schulze PC	2003	Germany	53	11	7.72 ± 0.9	6.36 ± 1.35	Patients (aged 71 ± 6 years) with chronic HF as a result of dilated cardiomyopathy or ischemic heart disease were included in this study.	A total of 11 age-matched patients (aged 61 ± 4 years), who were admitted with non-specific chest pain for the exclusion of coronary artery disease, served as controls.

McEntegart MB	2007	UK	30	7	11.57 ± 4.98	11.1 ± 4.225	Patients (aged 56.6 ± 1.3 years) with HF as a result of coronary artery disease.	Healthy controls (aged 60.4 ± 4.0 years) were recruited from patient companions at the cardiology clinics. In addition to age-matching a lifestyle history was taken to activity-match healthy controls to the patients.

Fernandes F	2007	Brazil	14	30	1.4 ± 0.8	8.1 ± 7.8	Patients (aged 47.8 ± 9.1 years) with HF as a result of Chagas’ disease.	The control group consisted of 30 healthy individuals (aged 43.9 ± 5.9 years).

Patel JV	2007	UK	101	110	7.90 ± 3.42	6.30 ± 2.67	The study group consisted of South Asian and European Caucasian descent patients with mild to moderate chronic HF.	Age, gender and ethnically matched controls were recruited from relatives of patients, healthy hospital staff, and local community groups.

Sierra-Johnson J	2008	USA	135	106	13.2 ± 12	7.6 ± 6	One hundred thirty-five consecutively eligible white patients (aged 55.8 ± 11 years) with a diagnosis of stable noncachectic systolic HF were recruited. The etiology of HF was ischemic or nonischemic dilated cardiomyopathy.	The control group consisted of 104 healthy individuals (aged 53.8 ± 15 years).

Straburzyńska-Migaj E	2010	Poland	41	8	9.2 ± 7.5	2.9 ± 1.2	The study group consisted of 41 patients (aged 50.1 ± 9.3 years) with HF. Coronary artery disease was diagnosed in 16 patients and dilated cardiomyopathy in the remaining 25 patients.	The control group consisted of eight healthy volunteers (aged 43.6 ± 14.7 years) of a similar age and gender distribution to the patients with HF.

Schulze PCMP	2011	USA	80	21	8.67 ± 5.45	3.7 ± 2.7	A total of 44 patients (aged 61 ± 12 years) with acute decompensated HF, and 26 patients (aged 63 ± 12 years) with chronic HF were enrolled in this study.	The control group consisted of 21 healthy individuals (aged 57 ± 13 years).

Bobbert P	2012	Germany	104	16	13.38 ± 16.53	7.34 ± 5.7	A total of 52 patients (aged 48.3 ± 13.3 years) with non-ischaemic dilated cardiomyopathy, and 52 patients (aged 49.3 ± 14.3 years) with inflammatory cardiomyopathy were enrolled in this study.	The control group was comprised of patients (aged 40.4 ± 7.7 years) admitted to the hospital for evaluation of suspected cardiomyopathy, in whom the diagnostic work-up finally revealed that their complaints were noncardiac in origin.

Karayannis G	2013	Greece	57	64	9 ± 10.3	8.7 ± 8.7	The study group consisted of 57 consecutive patients (aged 68 ± 12 years) hospitalized for decompensated HF.	The control group was comprised of 64 age- and sex-matched asymptomatic individuals (aged 64 ± 11 years).

Loncar G	2013	Serbia	73	20	7.3 ± 6.7	5.9 ± 3.2	The study group consisted of 73 males (aged 68 ± 7 years) with chronic HF due to ischaemic or idiopathic dilated cardiomyopathy.	The control group consisted of 20 male volunteers (aged 67 ± 7 years), without the history of any chronic disease and without any regular medication regimen.

Faxen UL	2017	Sweden	163	71	19.17 ± 26.02	10.8 ± 2.6	The study group consisted of 163 patients with HF.	The control group consisted of 71 individuals without self-reported cardiovascular disease or known hypertension.

Shiina Y	2018	Japan	46	12	10.1 ± 8.14	10.3 ± 3.5	The study group consisted of 46 patients (aged 32.1 ± 7.4 years) with HF due to congenital heart disease.	Twelve age-matched healthy subjects (aged 31.5 ± 7.2 years), all of whom were medical staff at our hospital and were enrolled as controls.

Dabarian AL	2019	Brazil	31	15	5.28 ± 4.45	5.61 ± 5.96	A total of 15 patients (aged 42.67 ± 6.72 years) with Chagas heart disease, and 16 patients (aged 46.2 ± 8.12 years) with idiopathic dilated cardiomyopathy were enrolled in this study.	Healthy controls (aged 48.3 ± 7.48 years) were matched to HF patients for age, sex, and body mass index.


HF, heart failure.

### Results of meta-analysis

**Figure 2:**
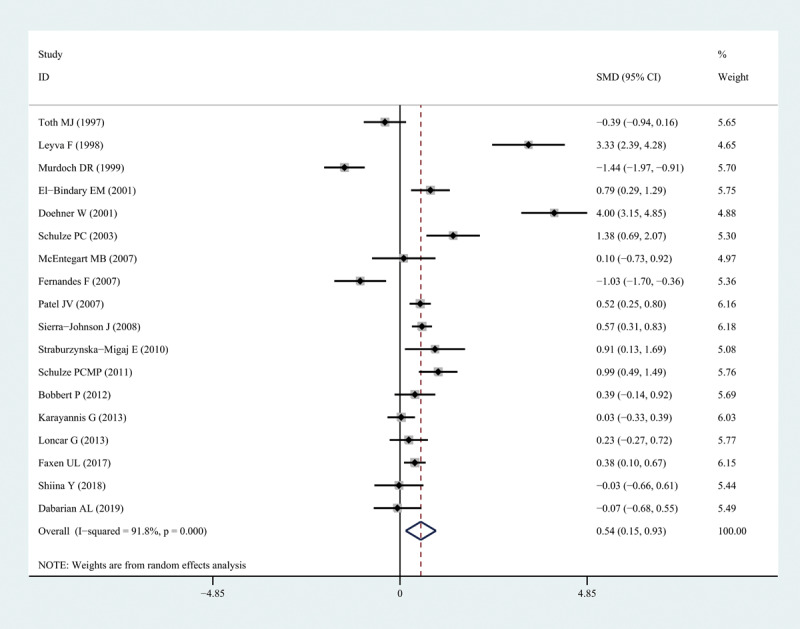
Forest plots of leptin level in patients with heart failure compared to healthy individuals. Diamond represents the pooled SMDs at 95% CI. SMD, standardized mean difference; CI, confidence interval.

Leptin levels in patients with HF were significantly higher than those in healthy individuals (SMD: 0.54, 95% CI [0.15, 0.93]; *I*^2^ = 91.8%, *P* < 0.05). Forest plots of the results are shown in Figure 2, and funnel plots are presented in Figure S1. We noted that the increase in leptin levels was more pronounced in patients with severe HF. However, because few studies on leptin levels in patients with different degrees of HF were available, we were unable to draw accurate conclusions.

### Sensitivity analysis and publication bias

**Figure 3:**
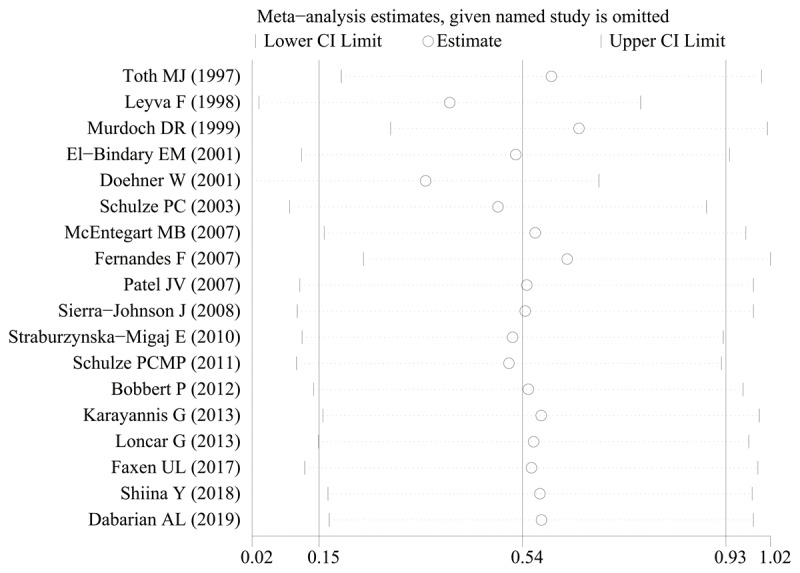
The sensitivity analysis results of leptin level in patients with heart failure compared to healthy individuals.

To ensure the reliability of the results, the calculations were recalculated by stepwise elimination of ≥1 studies and sensitivity analysis using a random-effects model. There was no significant change in the effect size after stepwise deletion of individual studies, indicating that the results were stable and reliable (Figure 3). A thorough and comprehensive database search was performed. Tests for the presence of publication bias in the results of systematic reviews were performed. Begger’s and Egger’s tests were used to assess overall publication bias in the included studies, with no bias observed in any of the included studies.

## Discussion

This systematic review is the first comprehensive evaluation of leptin levels in patients with HF. Eighteen independent case-control studies were included in this meta-analysis, and results showed that leptin levels in patients with HF were significantly higher than those in healthy controls. Fully characterizing the role of leptin in HF will contribute to clinical recommendations.

In 1994, Zhang et al. (11) cloned the mouse obesity gene (ob gene) from the adipose tissue of genetically obese mice (ob mice) for the first time, and Isse et al. successfully cloned the human obesity gene leptin (33). Six types of leptin receptors (Ob-Rb) have been identified, including Ob-Ra, Ob-Rb, Ob-Rc, Ob-Rd, Ob-Re, and Ob-Rf, of which Ob-Rb is the only long and functional receptor responsible for signal transduction in leptin cells. Ob-Rb has also been found in cardiomyocytes, indicating that leptin can act on the heart (34). Recent studies showed that the serum leptin concentrations in obese people were significantly higher than those in normal-weight people (35). Leptin mainly exists in a free state in obese individuals, and free leptin is generally believed to have biological activity (36).

At present, most researchers believe that excessive activation of neuroendocrine cytokines is an important cause of ventricular remodeling in HF. Ventricular remodeling is a complex process involving many factors. Previous studies suggested possible causes of elevated leptin levels in patients with HF. Swan et al. (37) and Doehner et al. (38) found that patients with chronic HF had hyperinsulinemia and insulin resistance. Insulin directly promoted leptin release from the endoplasmic reticulum of adipocytes (39). Animal experiments confirmed that insulin increased the expression of leptin RNA in adipose tissue and promoted protein secretion. Both short- and long-term insulin administration increased leptin gene expression. In HF, cardiac output is significantly decreased, and surrounding tissues and organs are not sufficiently perfused, causing ischemia and hypoxia, which are critical stress signals in the body. Stress leads to increased plasma cortisol levels in patients with HF failure. In vitro experiments confirmed that glucocorticoids directly or indirectly affected leptin secretion. Glucocorticoids can indirectly increase neuropeptide Y content in the hypothalamus, promoting ob gene expression. Glucocorticoids can also cause insulin resistance, manifesting as increased insulin secretion; thus, high insulin levels indirectly cause an increase in leptin levels (40). The kidney is the main organ responsible for the clearance of leptin, and damage to glomerular filtration or renal tubular reabsorption caused by various diseases can affect the clearance of leptin from the body, leading to leptin accumulation (41). Insufficient renal perfusion and decreased filtration rate in HF may be important factors in increased leptin levels.

High blood levels of leptin have been used to predict the severity of heart diseases, including HF (42). However, other studies showed an inverse relationship between plasma leptin levels and left ventricular mass (43). More compelling results supporting the role of leptin have been obtained from experimental studies. Most of these studies demonstrated a hypertrophic or cardiomyocyte hyperplasia response following leptin administration, indicating a direct hypertrophic effect (44,45). Myocardial overexpression during ischemia and reperfusion enhanced myocardial remodeling, leading to increased dysfunction (46). Furthermore, leptin influences myocardial remodeling by stimulating myocardial fibrosis, further contributing to the development of HF (47). Cardiomyocytes may be a target of leptin in circulation, and Nickola et al. have shown that leptin inhibited muscle contractility (48) leading to cardiac hypertrophy, a beneficial adaptive response in case of myocardial damage. However, continuous cardiac hypertrophy can lead to cardiac remodeling and the development of HF (49). Dong et al. showed that the effect of leptin on myocardial contractility in isolated rat cardiomyocytes was significantly attenuated by the ET-1 receptor blockers BQ123 and BQ788, and NADPH oxidase inhibitors. These results suggested that leptin inhibits myocardial contractions possibly through the ET-1 receptor and NADPH oxidase pathways (50). Adiarto et al. observed plasma ET-1 levels and myocardial ET-1M RNA expression by feeding ob gene-null mice and wild-type mice a high-fat diet (51). The results showed that plasma levels of ET-1 and leptin, and the expression of myocardial ET-1 mRNA, increased in wild-type mice. However, plasma ET-1 and myocardial ET-1 levels in ob gene-deletion mice were lower than those in wild-type mice. These results suggest that leptin mediates the upregulation of ET-1 mRNA, causing an increase in ET-1 levels.

Researchers believe that leptin can be used as a target for the treatment of heart disease and, in recent years, they have searched for leptin antagonists or inhibitors of leptin synthesis. In a study on HF rats, Na et al. found that CPU86017 (p-chlorobenzyl tetrahydrogen small protein) reversed myocardial changes caused by leptin through the ET-1 pathway (52). These findings may provide a research direction in the search for leptin antagonists.

Zeidan et al. demonstrated that leptin significantly activated RhoA in neonatal rat ventricular myocytes, and that activation was inhibited by an OBR antibody. Additionally, the hypertrophic effect was blocked by the inhibitors C3 exoenzyme and Y-27632, of RhoA and ROCK, respectively. They attributed the ROCK-dependent hypertrophic effect of leptin to an increase in actin polymerization, as indicated by a decrease in the G/F-actin ratio owing to LIM kinase-dependent cofilin phosphorylation (53). Their study also indicated that intact caveolae played a crucial role in RhoA/ROCK-dependent leptin-induced cardiomyocyte hypertrophy. Leptin significantly increases the expression of caveolae in cardiomyocytes, as confirmed by molecular analyses and electron microscopy. It is worth noting that OBR is co-localized with caveolae, and disrupting caveolae with the cholesterol-chelating agent methyl-β-cyclodextrin completely blocked the pro-hypertrophic effect of leptin. Additionally, the pro-hypertrophic effects of leptin in NRVMs were linked to the specific translocation of p38 MAPK into the nucleus, which was dependent on RhoA and caveolae, as demonstrated by a significant reduction in p38 MAPK nuclear translocation (54).

Central leptin has a two-fold impact on heart health, possibly causing ventricular atrophy and enhancing cardiac function in the presence of PPARβ/δ signaling. The beneficial effects of leptin disappear with PPARβ/δ suppression, highlighting the significance of this pathway (55).

The present study has some limitations. First, the methods used to measure leptin levels varied among the included studies. Second, different causes of HF may affect leptin levels. Third, patient characteristics, such as body weight, may have affected the analysis of the relationship between leptin and HF. Therefore, additional high-quality studies, especially prospective study, are required to further investigate the role of leptin in HF. Consequently, the results of the present meta-analysis should be interpreted with caution. Fully characterizing the role of leptin in HF will significantly contribute to diagnosis and improved treatment in clinical recommendations and guidelines.

## Conclusion

To our knowledge, this systematic review is the first to evaluate the relationship between leptin and HF. Leptin levels were significantly higher in patients with HF. Fully characterizing the role of leptin in HF will significantly contribute to diagnosis and improved treatment.

## Data Accessibility Statement

The original contributions presented in the study are included in the article/supplementary material. Further inquiries can be directed to the corresponding author.

## Additional Files

The additional files for this article can be found as follows:

10.5334/gh.1434.s1 File S1. Preferred reporting items for systematic review and meta-analyses (PRISMA) checklist.

10.5334/gh.1434.s2 File S2. Search strategy in databases.

10.5334/gh.1434.s3 Figure S1. Funnel plots of leptin level in patients with heart failure compared to healthy individuals.
