# Supplementary material for: Relationship Between Leptin and Heart Failure: A Meta-Analysis
Source: Glob Heart. 2025 May 23;20(1):44. doi: 10.5334/gh.1434 (PMC12101114; doi:10.5334/gh.1434)
Supplement: File S2. — Search strategy in databases. [file gh-20-1-1434-s2.pdf]

PubMed: "heart failure"[Title/Abstract] AND "leptin"[Title/Abstract]

Web of science: (TS = (heart failure)) AND TS = (leptin)

Wiley online Library: "heart failure"[Abstract] AND "leptin"[Abstract]

Embase: ((‘heart failure’:ti,ab) AND (‘leptin:ti,ab).
