# Supplementary figures and images for: Relationship Between Leptin and Heart Failure: A Meta-Analysis
Source: Glob Heart. 2025 May 23;20(1):44. doi: 10.5334/gh.1434 (PMC12101114; doi:10.5334/gh.1434)

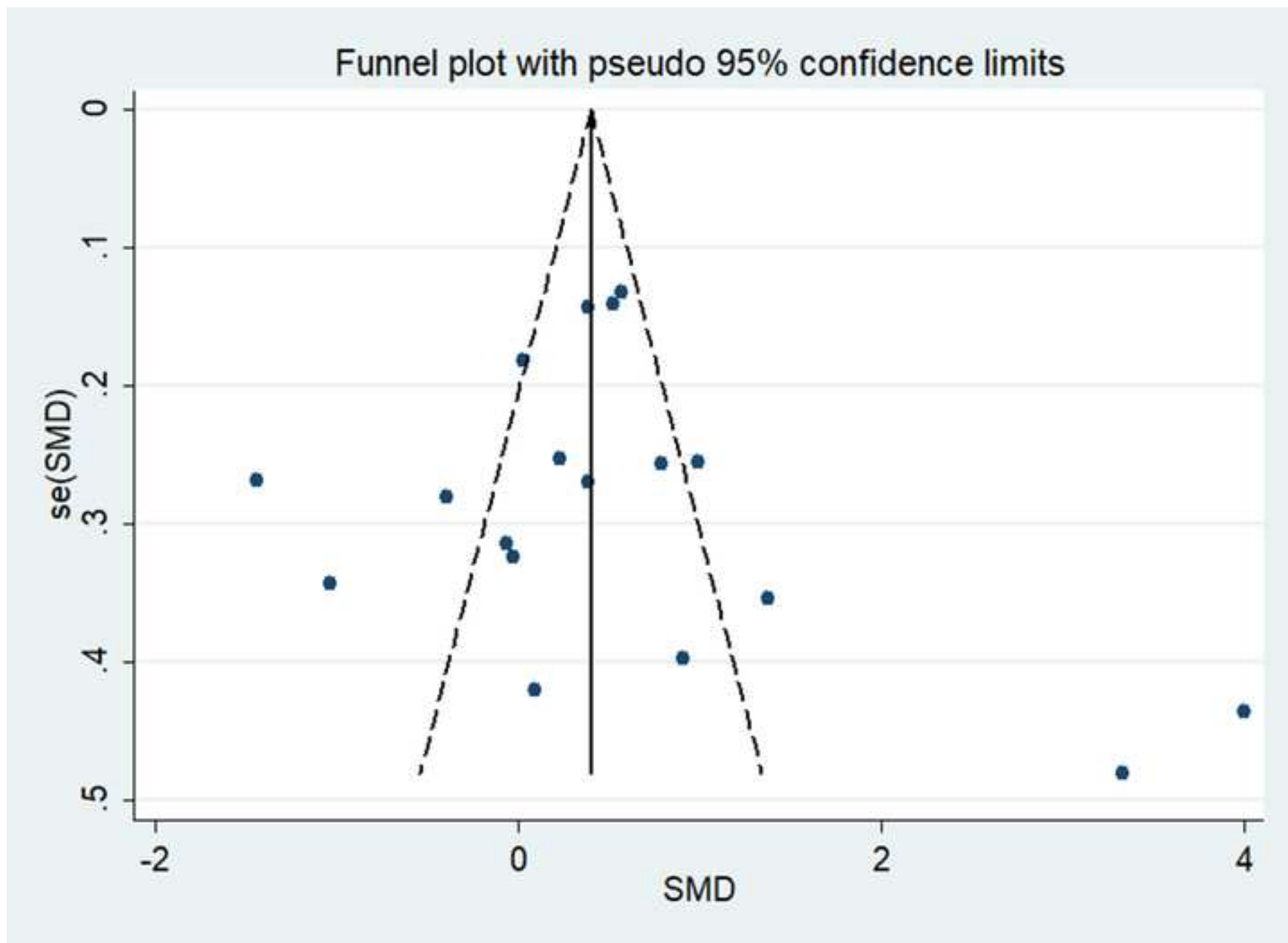

Supplement: Figure S1. — Funnel plots of leptin level in patients with heart failure compared to healthy individuals. [file gh-20-1-1434-s3.pdf]
